# Vat Photopolymerization-Fabricated Theranostic Hydrogels for Smart Wound Management

**DOI:** 10.3390/gels12050393

**Published:** 2026-05-02

**Authors:** Karl Albright Tiston, Laureen Ida Ballesteros, Jo Marie Venus Agad, Patrick Meracandayo, Karlos Mayo Silva, Toni Beth Lopez, Nadnudda Rodthongkum, Voravee P. Hoven, Rigoberto Advincula

**Affiliations:** 1 Department of Science and Technology, Metals Industry Research and Development Center, Taguig City 1631, Philippines; 2 Department of Chemistry, Faculty of Science, Chulalongkorn University, Bangkok 10330, Thailand; 3 Center of Excellence in Responsive Wearable Materials, Chulalongkorn University, Bangkok 10330, Thailand; 4 Department of Chemical and Biomolecular Engineering, University of Tennessee, Knoxville, TN 37996, USA

**Keywords:** 3D printing, vat photopolymerization, digital light processing, hydrogels, drug delivery, theranostics

## Abstract

Despite the demand for personalized wound care, integrating diagnostics and therapeutics into a unified platform remains a significant challenge. To address this, we developed a 3D-printed theranostic hydrogel using vat photopolymerization, enabling precise, multifunctional wound management. The hydrogel matrix, composed of poly(acrylamide-*co*-hydroxyethyl acrylate) and carboxymethyl cellulose, was chemically crosslinked with poly(ethylene glycol) diacrylate. Bromocresol purple was integrated into the photosensitive resin to enhance printing fidelity and serve as a diagnostic indicator, providing a distinct colorimetric shift upon skin infection. For controlled drug delivery, graphene oxide (GO) and levofloxacin were incorporated into the system. The 3D-printed hydrogel demonstrated superior swelling capacity (>600%), ideal for absorbing wound exudate. A semi-quantitative linear colorimetric response was observed across varying pH levels, allowing for clear differentiation between healthy healing skin (pH 4.0–6.0) and infected conditions (pH 7.0 and above). Furthermore, the hydrogel exhibited infection-stimulated therapy, with a cumulative levofloxacin release of 92.63% at pH 8, significantly higher than in acidic conditions. Moreover, the incorporation of GO further optimized the delivery profile by tuning absorption and release rates. Synergizing real-time monitoring and on-demand therapeutic action, this 3D-printed system offers a scalable, robust solution for future-ready, personalized wound management.

## 1. Introduction

Chronic wounds represent an escalating global health and socioeconomic challenge, predominantly affecting the elderly and individuals with diabetes, vascular diseases, or traumatic injuries [1,2]. Unlike acute wounds, chronic wounds, such as diabetic foot, venous, and pressure ulcers, fail to progress through the normal stages of tissue repair and are frequently complicated by persistent infection, prolonged inflammation, and impaired angiogenesis [3]. This kind of wound environment may render conventional passive dressings, thus necessitating more effective, active therapeutic interventions [4].

Current chronic wound management is primarily guided by the utilization of moisture-balancing dressings, targeted debridement, and advanced adjuncts such as negative-pressure wound therapy and biologic matrices to facilitate tissue repair [5]. However, these conventional interventions remain largely passive, relying on episodic clinical evaluations, standard cultures, and distinct diagnostic tests that fail to capture the dynamic physiological fluctuations of the healing process [6,7]. To address the limitations of separated therapeutic and diagnostic protocols, recent innovations are shifting toward “smart” wound dressings integrated with novel biosensing technologies [8,9,10]. By continuously tracking critical local biomarkers including pH, temperature, oxygenation, and bacterial burden via colorimetric or electrochemical sensors, these devices provide real-time, actionable data [9,10]. Ultimately, bridging the gap between passive treatment and active monitoring through fully integrated platforms capable of simultaneous diagnosis and therapy represents a critical frontier in optimizing chronic wound care.

While healthy skin and progressing, acute wounds maintain a protective, mildly acidic environment, chronic wounds are characterized by a sustained alkaline state with pH values often ranging from 7.4 to nearly 9 [7]. This prolonged alkalinity is driven by persistent inflammation and infection, which in turn delays healing by promoting bacterial proliferation and destructive protease activity [7,10]. Consequently, tracking this distinct shift toward higher pH serves as a highly valuable, non-invasive biomarker for continuously monitoring wound status and providing early detection of infection risk [6].

Traditionally, wound dressing has played an enormous role in wound management as these provide protective barriers for the wound to promote adequate healing. Wound dressing material ranges from fabrics such as cotton or viscose, polymers like polyurethane and silicone, and hydrogels [11]. Hydrogels have emerged as highly effective materials for advanced wound dressings due to their biocompatible, hydrated polymer networks that excel at localized, sustained drug delivery [3,12]. Capable of encapsulating a diverse array of therapeutics, ranging from small-molecule antibiotics to sensitive biologics like extracellular vesicles, hydrogels improve treatment efficacy directly at the wound site while minimizing systemic side effects [13,14]. Furthermore, the development of “smart” hydrogels allows for highly tailorable, stimuli-responsive systems that react to specific wound microenvironmental cues, such as the elevated pH or high reactive oxygen species (ROS) levels characteristic of chronic infections, to trigger on-demand drug release [15,16]. While their structural adaptability and responsive therapeutic capabilities make hydrogels exceptionally well-suited for treating complex wounds, current designs primarily function as passive therapeutic reservoirs, leaving a crucial gap for integrating real-time diagnostic feedback.

By combining therapeutic and diagnostic capabilities, the concept of “theranostics” arises as an integrated mechanism for smarter, simplified biomedical devices [17]. In this aspect, theranostic hydrogels represent a transformative approach to wound management by seamlessly integrating real-time diagnostic monitoring with localized, on-demand therapeutic delivery within a single “sense-and-respond” platform [17,18,19]. Unlike conventional materials, these smart dressings actively track critical physiological biomarkers such as pH, temperature, and specific biochemical cues, through embedded colorimetric, optical, or electrochemical sensors [6,19]. In response to these real-time inputs, such as an alkaline shift indicative of infection, the hydrogel matrix can trigger the targeted release of appropriate interventions, including antibiotics, anti-inflammatory agents, or regenerative biomolecules [13,15,19,20]. By bridging the gap between continuous physiological monitoring and adaptive drug delivery, theranostic hydrogels enable earlier detection of complications, reduce the need for frequent dressing changes, and provide a highly customized, dynamic healing environment for chronic wounds [17,18,19].

While hydrogels are highly effective platforms for localized wound management, fine-tuning controlled drug release remains a considerable problem. To mitigate this limitation, various functional additives, such as metal nanoparticles, mesoporous silica, and carbon nanotubes, are frequently incorporated into the hydrogel matrix to modulate delivery profiles. Among these, graphene oxide (GO) has emerged as an exceptional nanocarrier. The two-dimensional planar structure and immense surface area of GO allow exceptionally high drug-loading capacity, which mitigates proper dosage delivery [21]. GO also contains a highly functionalized amphiphilic surface which allows suitable drug and polymer network interactions. The unoxidized hydrophobic basal plane stably binds aromatic therapeutic agents, while abundant hydrophilic oxygen-containing functional groups ensure excellent compatibility with the surrounding hydrogel network [22,23]. Furthermore, GO controls drug delivery through specific non-covalent interactions, primarily π–π stacking, and can be disrupted through localized environmental triggers such as pH changes [22,24,25]. These specific interactions make GO an excellent candidate for fine-tuning drug delivery in hydrogel-based platforms.

To fully realize the potential of dynamic theranostic platforms, additive manufacturing (AM) offers a highly versatile approach for fabricating precise, patient-specific hydrogel geometries directly from digital models [26,27]. By building constructs layer-by-layer, AM allows for the meticulous adjustment of scaffold dimensions, pore distribution, and mechanical stiffness to match user needs [28,29]. Currently, direct ink writing (DIW) is the most widely used 3D printing technique for hydrogels; however, this extrusion-based method relies heavily on the shear-thinning behavior of continuous inks and often requires support materials or post-processing to maintain shape fidelity [30]. Consequently, DIW is inherently constrained by the scale of its extruded filaments, making the precise control necessary for fine micro-architectures challenging [31,32]. To overcome these structural limitations, this study utilizes vat photopolymerization (VPP), specifically digital light processing (DLP), to engineer the proposed theranostic hydrogel dressings. By employing patterned light to selectively cure photosensitive resins, DLP achieves exceptionally high print resolution and smooth surface finishes that are difficult to replicate with DIW [33,34]. More importantly, this light-based technique excels at generating complex, intricate internal geometries, such as compartmentalized constructs, microlattices, and macropore structures [35,36]. From a drug delivery perspective, these advanced architectures are crucial. These significantly increase the functional surface area, modulate internal diffusion pathways, and allow for locally varied crosslink densities [37,38,39]. Ultimately, leveraging DLP to integrate such structural complexity empowers the hydrogel dressing with highly programmable release kinetics, seamlessly linking sophisticated physical design with advanced, localized wound therapy.

Herein, we report a high-fidelity, DLP 3D-printed hydrogel platform for the simultaneous sensing and treatment of wound infections. We have formulated a semi-interpenetrating polymer network made from a carboxymethyl cellulose-based matrix crosslinked with a multicomponent poly(acrylamide-*co*-hydroxyethyl acrylate-*co*-polyethylene glycol diacrylate), poly(AAm-co-HEA-co-PEGDA). Notably, bromocresol purple was utilized as a dual-functional additive, serving both as a photoabsorber to ensure superior printing resolution and as a real-time colorimetric biosensor for pH-based infection diagnostics. Beyond sensing, the platform provides autonomous therapeutic intervention as the loaded antibiotic, levofloxacin, exhibited accelerated release at pH above 7.0 via diffusion. Moreover, graphene oxide (GO) was also added to the formulation to further modulate drug release, ensuring a more controlled and sustained delivery of the therapeutic agent. Lastly, we demonstrated that the advanced spatial resolution of 3D printing enables the precise fabrication of customized macroporous architectures, which later provided further control in drug release. By deliberately engineering these internal diffusion pathways and optimizing the overall surface area-to-volume ratio, the system achieves highly tunable and inherently more efficient drug release. This multifunctional system represents a versatile, scalable advancement in the design of next-generation “smart” wound management materials.

## 2. Results and Discussion

### 2.1. Formulation of Photopolymerizable Resin and DLP 3D Printing of Hydrogels

**Figure 1:**
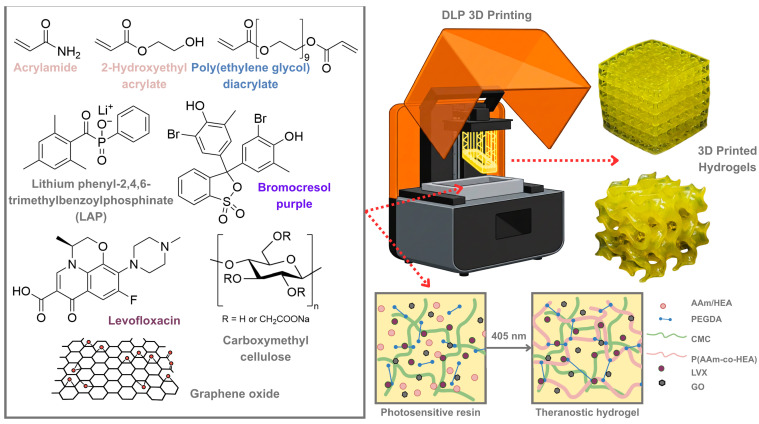
Schematic diagram showing the DLP 3D printing of a theranostic hydrogel for wound infection, including the hydrogel precursors and polymer network formation process.

The preparation of the photopolymerizable hydrogel resin was done by dissolving acrylamide (AAm), 2-hydroxyethyl acrylate (HEA), polyethylene glycol-9 (PEGDA), carboxymethyl cellulose (CMC), Lithium phenyl-2,4,6-trimethylbenzoylphosphinate (LAP), graphene oxide (GO), levofloxacin (LVX), and bromocresol purple (BCP) in distilled water (Figure 1). The resulting solution was used as a photosensitive resin in a DLP 3D printer. CMC is a cellulose-derived water-soluble biopolymer added to hydrogel formulation for mechanical reinforcement and increased hydrophilicity, suitable for wound dressing applications [40,41]. The hydrogel was formed after exposing the liquid resin precursor to 405 nm light where the LAP photoinitiator decomposes and produces radicals, initiating the photopolymerization. The main polymer network is made from poly(AAm-co-HEA) crosslinked with PEGDA. Overall, a semi-interpenetrating polymer network (semi-IPN) hydrogel was formed upon printing. The pH-responsive dye, BCP, acts as the photoabsorber in the resin formulation, which inhibits further photopolymerization in previously printed layers, thus allowing higher-resolution prints [42,43]. BCP acts similarly to the more commonly used tartrazine dye, as both afford a yellow hue upon dissolution in the photopolymerizable resin [44]. In this case, BCP has a dual function of acting as a photoabsorber, improving the printing fidelity, and later as a pH-responsive dye for potential infection monitoring. Ultimately, the successful combination of these constituents results in a robust material system that balances mechanical reinforcement with precise printability. This approach ensures that the final hydrogel construct possesses the necessary structural and functional properties required for complex biomedical devices, such as 3D-printed smart wound dressings.

**Figure 2:**
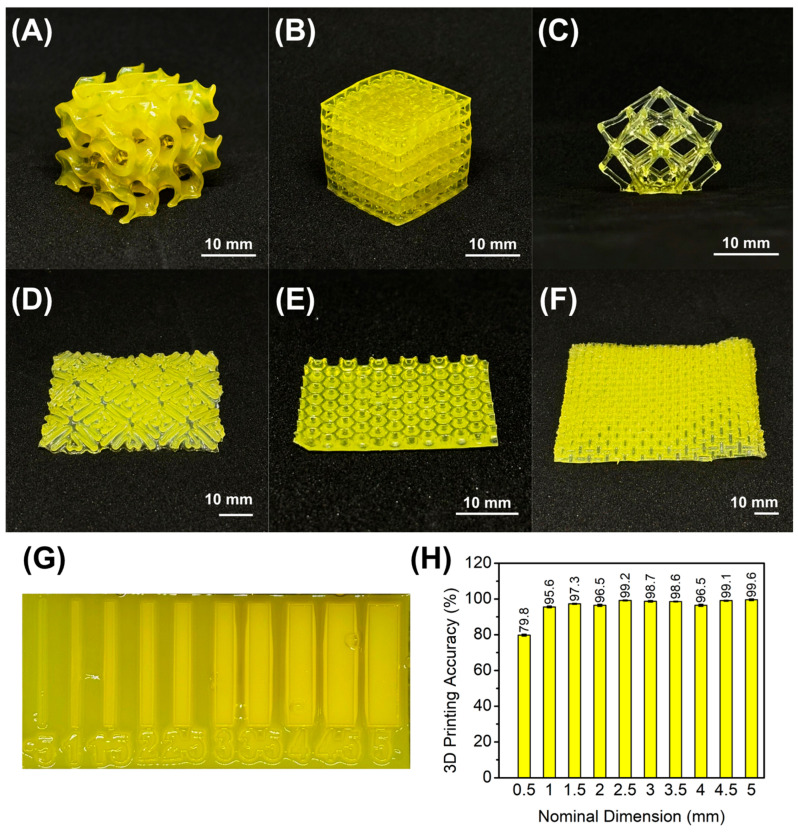
3D-printed poly(AAm-co-HEA-co-PEGDA)/CMC/GO hydrogels: (**A**) Gyroid cube, (**B**) lattice cube, (**C**) torture specimen, (**D**) auxetic mesostructured dressing, (**E**) honeycomb dressing, (**F**) auxetic grid dressing; (**G**) 3D-printed wall calibration specimen; (**H**) 3D printing accuracy.

To demonstrate the 3D printability of the formulated hydrogel resins, various structures were fabricated using the Creality Halot One, a commercial DLP 3D printer with 2K resolution. Architectures with complex motifs were printed to showcase the resin capabilities, including a gyroid infill, a square grid, a lattice calibration specimen, and various patterned wound dressing prototypes (Figure 2A–F). Vat photopolymerization enables the creation of intricate patterns, such as lattices and hollowed structures, which are often difficult to achieve with extrusion-based techniques [42,45,46]. Consequently, applying DLP 3D printing to theranostic hydrogels facilitates the fabrication of smart wound dressings with personalized designs. After printing, the hydrogels were quickly rinsed with distilled water to remove excess resin and then exposed to 405 nm light to complete photopolymerization. Printing fidelity was assessed by comparing the dimensions of a 3D-printed thickness calibration sample to the nominal dimensions (Figure 2G). While the printed hydrogels generally exhibited high accuracy (Figure 2H), walls with the finest dimensions (0.5 mm) were larger than the nominal value. This discrepancy is likely attributable to gel swelling during the printing process. Additionally, the incorporation of BCP effectively prevented overexposure by absorbing excess UV light and controlling the depth of cure (Figure S1, Supporting Information). Overall, the 3D-printed constructs demonstrated suitable dimensional accuracy, confirming the high printing fidelity of the hydrogel resin.

### 2.2. Characterization of the 3D-Printed Hydrogels

**Figure 3:**
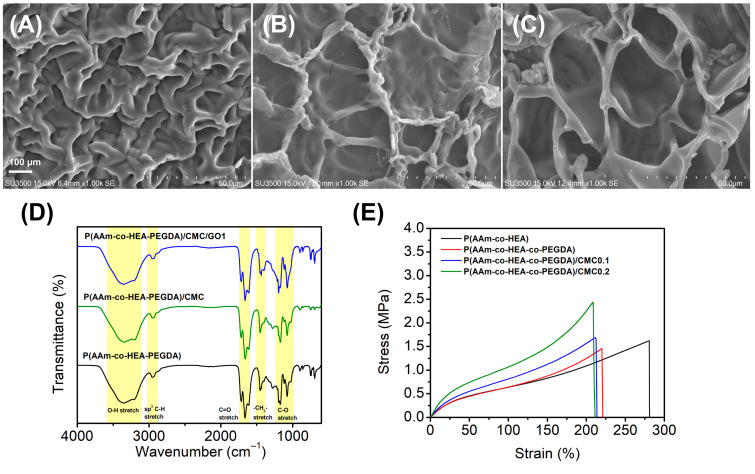
SEM images of (**A**) poly(AAm-co-HEA-co-PEGDA), (**B**) poly(AAm-co-HEA-co-PEGDA)/CMC, (**C**) poly(AAm-co-HEA-co-PEGDA)/CMC/GO; (**D**) FT-IR spectra of selected 3D-printed hydrogel samples; (**E**) tensile stress–strain curves of selected 3D-printed hydrogel samples.

Scanning electron microscopy revealed porous structures for the formulated hydrogel (Figure 3A–C). The base copolymer, poly(AAm-co-HEA-co-PEGDA), exhibits a relatively dense and tightly packed polymeric network with irregularly shaped, smaller pores. Upon the incorporation of CMC to form poly(AAm-co-HEA-co-PEGDA)/CMC, the microstructure transitions into a more defined, interconnected, and distinctly open porous architecture, suggesting that the CMC chains aid in organizing and stabilizing the polymer matrix [47]. The subsequent incorporation of graphene oxide (GO) transforms the morphology into a regular, macroporous, honeycomb-like architecture. These significantly enlarged and well-defined pores suggest that the dispersed GO nanosheets function as a reinforcing agent within the pore walls, preventing structural collapse and facilitating highly interconnected porosity [48]. Good gel porosity is highly advantageous for exudate absorption, improved drug diffusion and gas exchange in wound management applications.

Fourier transform infrared (FT-IR) spectroscopy was utilized to characterize the chemical structures and verify the successful integration of components within the hydrogel networks (Figure 3D). Across all formulations, the spectra exhibit characteristic absorption bands that confirm the expected functional groups. The broad band centered around 3400 cm^−1^ corresponds to the overlapping O-H and N-H stretching vibrations, reflecting the presence of hydroxyl and amine groups from the primary monomers, alongside contributions from CMC and GO. The absorption at 2960 cm^−1^ is attributed to sp^3^ C-H stretching, while the prominent peak at around 1700 cm^−1^ confirms the C=O stretching of the constituent amide and ester groups. Additionally, the peaks at 1460 cm^−1^ and 1160 cm^−1^ represent -CH_2_- bending and C-O stretching vibrations, respectively. Moreover, only a slight widening of peaks around 1460 cm^−1^ and 1160 cm^−1^ was observed in the spectrum of the hydrogel containing 1 ppm GO. This might be due to the GO interaction with the polymer matrix [49]. Aside from this observation, significant changes in the peaks after the addition of 1 ppm GO were not evident as this amount typically falls below the instrument’s limit of detection [50,51]. Furthermore, any weak signals from the trace graphene oxide would be entirely masked by the overlapping infrared absorption bands of the host polymer matrix [51,52]. The overall similarity of the spectral profiles upon the successive addition of CMC and graphene oxide suggests that the fundamental polymeric backbone is preserved, indicating that the composite materials are primarily stabilized through robust physical interactions, such as intermolecular hydrogen bonding, rather than alterations to the covalent network

The tensile mechanical properties of the hydrogels were evaluated to understand the structural impact of the crosslinker and carboxymethyl cellulose (CMC) on the polymer matrix (Figure 3E). The base copolymer, poly(AAm-co-HEA), exhibited the highest elasticity with an elongation at break of 281%, coupled with a tensile strength of 1.623 MPa. The introduction of PEGDA to form poly(AAm-co-HEA-co-PEGDA) resulted in a tighter crosslinked network, which predictably restricted chain mobility, reducing the maximum strain to 221%, with tensile strength of 1.456 MPa. Most notably, the incorporation of CMC into the hydrogel system served as an effective reinforcing agent. As the CMC concentration increased from 0.1 to 0.2 wt%, the ultimate tensile stress significantly enhanced, peaking at nearly 2.440 MPa for poly(AAm-co-HEA-co-PEGDA)/CMC0.2. While this reinforcement came with a marginal trade-off in stretchability, the dramatic increase in stiffness and tensile strength suggests that the rigid CMC chains successfully established strong intermolecular interactions with the primary polymer network, effectively transferring stress and improving the overall mechanical robustness of the composite hydrogel [53]. Ultimately, this synergistic balance of enhanced tensile strength and sustained elasticity makes the CMC-reinforced hydrogels highly promising for wound dressing applications, ensuring conformation to dynamic body movements while resisting mechanical failure [30].

### 2.3. Swelling Behavior of the 3D-Printed Hydrogels

**Figure 4:**
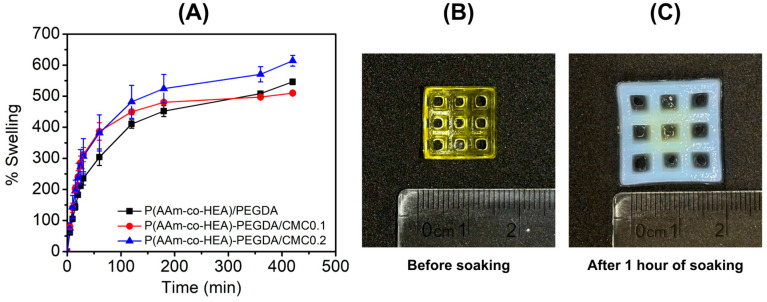
Swelling behavior of 3D-printed hydrogels. (**A**) Percentage of swelling vs. time. Change in 3D-printed hydrogel size (**B**) before soaking (**C**) after soaking.

The water absorbance of the formulated hydrogels was assessed by determining the swelling behavior of representative formulations. Dried 3D-printed specimens were soaked in pH 7.4 PBS and changes in weight were recorded. Figure 4 illustrates the time-dependent swelling percentage over a period of roughly seven hours, revealing that all three compositions follow a characteristic kinetic profile: a rapid initial uptake of water followed by a gradual plateau as the materials approach equilibrium. This rapid water uptake can be attributed to the previously discussed porous structure of the hydrogel. Moreover, the hydrogel containing 0.2 wt% CMC demonstrates the superior swelling capacity throughout the entire duration, achieving the highest maximum swelling of approximately 610%. This suggests that the higher concentration of CMC introduces sufficient hydrophilic groups and ionic charges to maximize the osmotic pressure within the network, driving substantial water absorption [53,54,55]. The addition of 0.1 wt% CMC also significantly increased initial water uptake as compared to the control formulation. Interestingly, the water uptake eventually plateaued at a slightly lower or comparable level (513%) to the control (536%) by the end of the experiment. This indicates that while small amounts of CMC accelerate the kinetics of hydration, they do not necessarily expand the ultimate equilibrium volume as effectively as the 0.2 wt% loading. Aside from CMC, the high water uptake can also be attributed to the addition of GO to the formulation. This is driven by the interaction between water and the hydrophilic sites on GO, specifically the –OH groups, which foster hydrogen bonding and enhance the diffusion and perfusion of water into the matrix [23,56,57]. Such high swelling capabilities are particularly advantageous for wound dressing applications, where the ability to absorb excess exudate while maintaining a moist healing environment is essential [57,58]. Furthermore, the significant swelling of these hydrogels is essential for the sustained release of encapsulated therapeutic agents [57]. Their exceptional capacity for biofluid absorption positions these synthetic bioactive hydrogels as highly promising candidates for advanced wound healing applications.

### 2.4. Colorimetric Response of 3D-Printed Hydrogels

**Figure 5:**
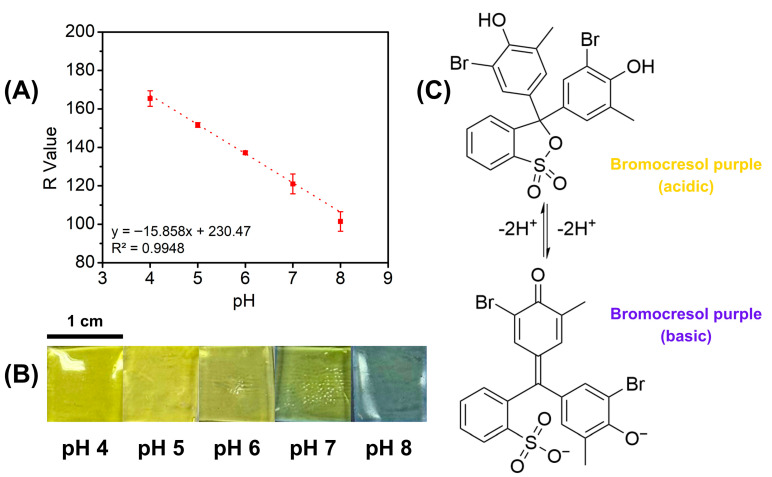
(**A**) Hydrogel red color value vs. pH, (**B**) color change of the hydrogel, (**C**) change in bromocresol purple structure due to pH shift.

To assess the colorimetric response of the 3D-printed hydrogels, 3D-printed hydrogel patches were immersed in solutions of varying pH (pH 4–8) for one minute. This procedure was designed to simulate the pH variations associated with wound infection. The 3D-printed hydrogels exhibited an initial yellow hue, attributed to the presence of bromocresol purple (BCP) in the resin. A significant color shift was observed at pH 6, where the hydrogel transitioned to a slight green. At pH 7, this green coloration became more pronounced, and at pH 8, the hydrogel ultimately shifted to blue (Figure 5B). These observations align with the expected behavior of BCP, which remains yellow below pH 5.2 and transitions to purple above pH 6.8 [59]. Consequently, this study demonstrates the dual functionality of BCP, acting as a photoabsorber during DLP 3D printing and serving as a pH-responsive indicator for wound infection monitoring. In a clinical setting, the gel functions by reacting to the bacterial metabolites, which typically shift the wound environment from acidic to alkaline as an infection develops. As the wound fluid permeates the porous hydrogel matrix, the embedded BCP molecules undergo a structural rearrangement (Figure 5C) that alters their light absorption, providing a real-time visual warning through a clear color change from yellow to purple.

While visual inspection allows for a rapid qualitative assessment of the significant color shifts associated with pH increases, a quantitative analysis was performed to rigorously validate these variations and construct a calibration curve. To generate a semi-quantitative model for colorimetric response, digital images of the hydrogels were processed using ImageJ software according to previously described methods [60]. Data points representing the red, green, and blue (RGB) channels, along with grayscale intensities, were extracted and correlated with specific pH values. The resulting plots demonstrated a highly linear relationship between color intensity and pH level. Specifically, the red (R) channel yielded the highest correlation coefficient (R^2^ = 0.9948, Figure 5A), closely followed by the green channel (R^2^ = 0.9919, Figure S2, Supporting Information) and grayscale values (R^2^ = 0.9675, Figure S3, Supporting Information). This strong linearity confirms the reliability of the formulation as a sensor for monitoring infection progression. Furthermore, this correlation establishes a framework for smart wound-monitoring systems that integrate qualitative visual inspection with smartphone-based digital colorimetry. This method permits the non-invasive, real-time measurement of wound status, effectively connecting patient self-care with professional clinical supervision to ensure prompt and suitable medical treatment.

### 2.5. In Vitro Drug Release Study

In this study, we investigated the influence of two primary factors on drug release kinetics: GO content and 3D-printed structural geometry. Levofloxacin (LVX) was used as the model drug, with release profiles monitored via UV-Vis spectrophotometry. To simulate the dynamic wound microenvironment, drug release was evaluated under conditions mimicking healthy skin (pH 5.0), physiological fluid (pH 7.4), and infected tissue (pH 8.0). The cumulative release data demonstrated clear pH-responsive behavior, where drug release increased significantly with rising pH. The lowest release was observed at pH 5.0, followed by moderate release at pH 7.4, with the maximum release occurring at pH 8.0. This trend is characteristic of carboxylated hydrogels, such as the CMC and CMC/GO matrix used in this system [61,62,63]. As the environment becomes more alkaline, the carboxylic acid groups (-COOH) within the polymer network deprotonate into negatively charged carboxylate ions (-COO^−^). The resulting electrostatic repulsion between polymer chains causes the hydrogel network to swell and expand, thereby increasing the pore size and facilitating faster drug diffusion [63]. This behavior aligns well with the physiological requirements of wound management. The minimal release at pH 5.0 helps prevent premature drug loss on healthy skin. However, as the wound environment shifts towards the alkaline pH typical of bacterial infection (pH 7.4–8.9), the hydrogel’s swelling response triggers a significant increase in levofloxacin release. This infection-responsive mechanism ensures that the highest therapeutic dose is delivered precisely when bacterial proliferation is most aggressive, optimizing antibacterial efficacy while minimizing unnecessary exposure in non-infected states.

**Figure 6:**
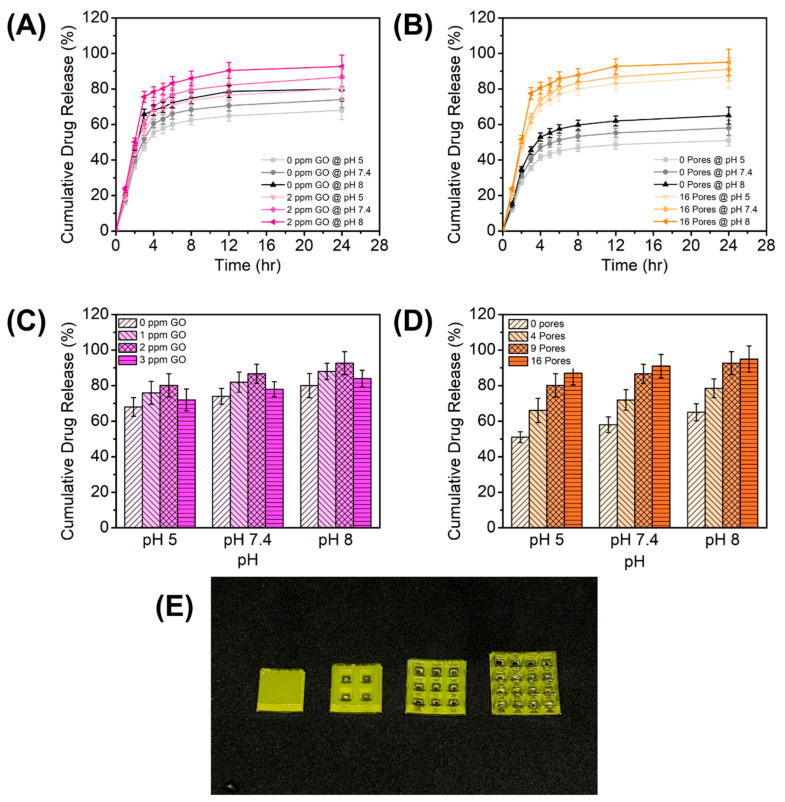
Drug release profile: (**A**) Effect of 0 vs 2 ppm GO, (**B**) effect of 0 vs 16 pores structure. Total cumulative drug release: (**C**) Effect of GO, (**D**) effect of number of pores. (**E**) Weight-normalized 3D-printed gel pads with different numbers of pores (0, 4, 9, 16 pores).

Comparing the drug release of 3D-printed hydrogels with and without GO, higher drug release was consistently seen with hydrogels with GO than those without GO (Figure 6A). To test the effect of GO, a standard nine-pore gel pad was 3D-printed and subjected to a drug release experiment. Among the concentrations tested, the 2 ppm GO formulation exhibited the highest cumulative drug release (pH 8) at 92.63%, followed by 1 ppm GO at 88.01%. Interestingly, increasing the concentration further to 3 ppm GO resulted in the lowest drug release among the GO-loaded samples, at 83.98% (Figure 6C). This non-linear trend suggests an optimal dispersion threshold at 2 ppm. At this concentration, the hydrophilic functional groups on the GO sheets likely enhance water uptake and matrix swelling, facilitating drug diffusion [64,65]. However, at 3 ppm, the excess GO nanosheets likely undergo π-π stacking and agglomeration [66]. These aggregates reduce the effective surface area and create tortuous physical barriers within the hydrogel network, thereby hindering the release of the drug [66,67]. Nonetheless, the observed behavior suggests that the GO nanosheets act as structural spacers, potentially interrupting the polymer chain packing to create a more permeable network for drug diffusion [68]. Additionally, the superior surface-to-volume ratio of GO facilitates high-capacity drug anchoring and release, governed by the dynamic interplay between the drug molecules and the graphene surface [68,69]. The observed enhancement can also be attributed to the high affinity between LVX and the GO surface, mediated by two primary mechanisms: π-π stacking between the GO lattice and the fluoroquinolone rings of LVX, and hydrogen bonding between the GO’s oxygen-containing groups and the drug’s amine moieties [20]. Furthermore, the release profile is governed by the pH-dependent ionization of these functional groups. Variations in pH alter the electrostatic interplay between the drug and the filler, thereby tuning the adsorption strength and controlling the release rate [66,68,70]. From the results, the hydrogel containing GO consistently demonstrated a significantly higher cumulative drug release compared to the GO-free formulation. Mechanistically, we hypothesize that GO acts as a high-capacity reservoir within the hydrogel matrix [21]. During loading, LVX is strongly adsorbed onto the GO surface via π-π stacking between the aromatic rings of GO and the fluoroquinolone structure of LVX, alongside hydrogen bonding between the oxygen-containing functional groups (hydroxyl and carboxyl) of GO and the amine groups of LVX [65]. The enhanced, pH-dependent release profile is driven by the alteration of these non-covalent interactions. As the environment becomes more alkaline, mimicking infected wound exudate, the ionization states of both LVX and the functional groups on GO shift [71]. This induces electrostatic repulsion and weakens the drug–nanomaterial interactions, facilitating rapid diffusion of the drug from the matrix, with the maximum LVX release observed at pH 8. Ultimately, the strategic incorporation of GO creates a synergistic release mechanism, coupling the structural expansion of the polymer network with dynamic, pH-responsive chemical interactions to precisely govern the sustained delivery of the drug.

One advantage of additive manufacturing is that it allows the incorporation of specialized design and architecture that can further enhance the functionality of the printed object [30]. The drug release capability of the 3D-printed hydrogels was evaluated to understand the influence of structural design, specifically the inclusion of pore channels, on the release profile. As illustrated in Figure 6D, the introduction of porous channels into the hydrogel matrix had a profound impact on the total drug released. A clear positive correlation was observed between pore number and cumulative release percentage. The solid, bulk hydrogels (0 pores) consistently demonstrated the lowest drug release across all pH conditions, maxing out at approximately 65% release at pH 8. In contrast, the scaffolds designed with 16 pores achieved significantly higher release rates, reaching nearly 95% under the same conditions. It is critical to note that the total mass of the hydrogel was standardized across all samples, regardless of the pore count. Therefore, the observed increase in drug release is not a function of varying drug payloads or polymer content but is attributable strictly to the geometric modification. We hypothesize that the inclusion of macropore structures in the 3D-printed gel has increased the surface area available for drug diffusion, facilitating a more efficient mass transfer of the drug from the gel to the surrounding environment. Looking at the time-dependent release profiles (Figure 6B), the 16-pore scaffolds exhibited a rapid initial burst release within the first 4 h, followed by a sustained plateau. This rapid onset is advantageous for therapeutic applications requiring an immediate high dose to combat infection or inflammation. Conversely, the 0-pore bulk samples showed a much slower, more gradual release profile, struggling to release much of their payload within the 24 h window. These results underscore the distinct advantage of utilizing 3D printing technologies for pharmaceutical applications. Unlike traditional molding techniques, 3D printing allows for the precise, digital manipulation of scaffold geometry to increase drug delivery efficiency [72,73]. By simply modifying the CAD model to include 4, 9, or 16 pores, the drug release profile can be tuned to meet specific patient needs. A patient requiring rapid drug availability could be prescribed a highly porous 16-pore dressing, while a condition requiring slow, sustained release might benefit from a solid or low-porosity design. This ability to decouple geometric complexity from fabrication difficulty represents a significant leap forward in the design of personalized, tunable drug delivery systems.

### 2.6. Drug Release Kinetics

With the cumulative drug release data, the in vitro drug release profiles were mathematically modeled using the Higuchi, Korsmeyer–Peppas, and Peppas–Sahlin equations to elucidate the underlying transport mechanisms governing the release of the drug from the 3D-printed hydrogel scaffolds. The kinetic parameters obtained for hydrogels with varying GO concentrations (GO-0 to GO-3) reveal a release mechanism that is predominantly anomalous (non-Fickian) transport (Table 1). Across all formulations at pH 5 and 7.4, the release exponent *n* from the Korsmeyer–Peppas model consistently hovered around 0.69. This value, falling between 0.5 and 1.0, indicates that the drug release is driven by a coupling of two phenomena: Fickian diffusion of the drug through the hydrogel matrix and the relaxation (swelling) of the polymer chains [74]. Interestingly, the release kinetics demonstrated pH-sensitivity. At pH 8, *n* increased to values between 0.81 and 0.86. This shift towards Case II transport suggests that at higher pH, the contribution of polymer relaxation and potentially erosion becomes more significant compared to simple diffusion [74,75]. This is likely due to the increased ionization of the carboxyl groups in the CMC and polymer network at basic pH, leading to greater electrostatic repulsion, higher swelling ratios, and faster chain mobility [76]. In addition, dispersion of GO nanosheets creates a tortuous path for the drug molecules. Instead of moving in a straight line through the water-filled pores of the hydrogel, the drug molecules must navigate around the impermeable graphene sheets [77,78]. The Peppas–Sahlin model further corroborated this, showing mostly positive values for both the Fickian (*k*_1_) and relaxation (*k*_2_) constants, confirming that both mechanisms are operative and competitive.

The introduction of 3D-printed pores had a profound impact on both the rate and mechanism of drug release. As expected, the release rate increased substantially with pore density. For instance, at pH 8, the Higuchi rate constant *k_H_* nearly doubled from 24.52 for the non-porous control to 41.25 for the 16-pore scaffold (Table 2). This acceleration is directly improved by the increased surface area-to-volume ratio provided by the printed macro-channels, which facilitates faster solvent penetration and shorter diffusion path lengths for the drug [79,80]. More notably, the mechanism of release shifted drastically in the highly porous scaffolds. While the non-porous and low-porosity (4 pores) samples maintained anomalous transport characteristics (*n* ≈ 0.58–0.74), the high-porosity samples (9 and 16 pores) exhibited Super Case II transport kinetics (*n* > 0.89, reaching 1.05 at pH 8). An *n* value exceeding 0.89 indicates that the release is dominated by polymer relaxation and erosion rather than diffusion. The presence of macropores likely allows for rapid bulk water uptake, leading to immediate swelling stresses and accelerated degradation or macro-erosion of the hydrogel lattice [81,82]. This structural breakdown facilitates a rapid “dumping” of the drug, overpowering the diffusion-controlled regime.

Among the three kinetic models applied, the Peppas–Sahlin model consistently provided the best fit across all formulations and pH conditions, with R^2^ values exceeding 0.99. This superior fit is attributed to the model’s ability to decouple and quantify the individual contributions of Fickian diffusion (*k*_1_) and Case II relaxation (*k*_2_). The Korsmeyer–Peppas model also showed high applicability, whereas the Higuchi model, which assumes purely diffusion-controlled release from a non-swelling matrix, showed the poorest fit. This statistical comparison confirms that the swelling and relaxation of the 3D-printed hydrogel matrix are critical factors that cannot be ignored in the design of these drug delivery systems. Overall, these results indicate that both GO incorporation and scaffold porosity serve as critical handles for modulating the drug release mechanism, offering dual strategies to control the transition between diffusive and relaxational kinetics for optimized LVX delivery.

## 3. Conclusions

We report on the successful development of a novel vat photopolymerization 3D-printed hydrogel designed for simultaneous wound monitoring and therapeutic delivery. By integrating bromocresol purple directly into the resin, we achieved high-fidelity digital light processing (DLP) of customizable prototypes that function as high-sensitivity colorimetric sensors (R^2^ = 0.9948). The resulting scaffolds exhibit robust mechanical integrity (1.456 MPa) and high extensibility (221%), coupled with an optimized macroporous architecture that ensures effective exudate management. Furthermore, the synergistic incorporation of graphene oxide enables a sophisticated pH-responsive mechanism, achieving a 92.63% cumulative release of levofloxacin at infection-simulating alkaline levels (pH 8.0). Further development of this material can include other compatible therapeutics such as ciprofloxacin, gentamicin, moxifloxacin, and others with aromatic or ionizable groups. Moreover, testing with other buffer systems to simulate complex wound microenvironments, leading to in vivo testing, can be done. Overall, this dual-functional platform demonstrates immense potential for the scalable manufacturing of smart dressings, providing a versatile foundation for personalized, theranostic medical interventions.

## 4. Materials and Methods

### 4.1. Materials

Acrylamide (AAm), 2-hydroxyethyl acrylate (HEA), poly(ethylene glycol) diacrylate (n = approx. 9) (PEGDA), lithium phenyl-2,4,6-trimethylbenzoylphosphinate (LAP), and levofloxacin (LVX) were obtained from Tokyo Chemical Industry (Tokyo, Japan). Sodium carboxymethyl cellulose (~90 KDa) was purchased from SinoCMC (Qingdao, China). Bromocresol purple (BCP) was purchased from Techno PharmaChem (New Delhi, India). Graphene oxide (v20) was acquired from Standard Graphene (Ulsan, Republic of Korea).

### 4.2. Preparation of Photosensitive Resin and 3D Printing of Poly(AAm-co-HEA-co-PEGDA)/CMC/GO Hydrogels

A simple one-pot mixing was done to prepare the photosensitive hydrogel resin. The monomers, CMC, LAP, GO, LVX, and BCP were mixed with distilled water at room temperature until a clear solution was achieved. The hydrogel resins were stored in amber-colored glass vials and kept away from light. Before printing, the resins were sonicated to remove trapped gas and air bubbles. Detailed formulations are listed in Table 3. Various objects were then 3D-printed using a DLP 3D printer (Creality Halot One, Shenzhen, China). Printed gels were washed with distilled water to remove excess unpolymerized resin, then exposed to 6 W 405 nm UV light for 5 min to allow full curing.

### 4.3. Characterization Tests

Tensile tests of 3D-printed hydrogels were done using a universal testing machine (Instron 3343, Instron, Norwood, MA, USA). 3D-printed standard dogbone specimens were tested with a crosshead speed of 50 mm min^−1^ and a load cell of 500 N. All ultimate tensile strength and strain tests were performed in triplicate. Dimensional accuracy of the 3D printing process was assessed by printing a standard wall thickness structure, then measured by a coordinate measuring machine (Hexagon Optiv M, Hexagon Manufacturing Intelligence, Stockholm, Sweden). To check gel porosity, samples were observed under a scanning electron microscope (Hitachi SU3500, Hitachi, Tokyo, Japan) using 15 kV accelerating voltage at 12.5 mm working distance. Fourier transform infrared (FT-IR) spectra of representative hydrogels were obtained using an FT-IR spectrometer (Perkin Elmer Frontier, Waltham, MA, USA) with an attenuated total reflectance accessory.

### 4.4. Swelling Test

To determine the swelling capacity of the optimized formulation, a freeze-dried 3D-printed hydrogel was immersed in a phosphate-buffered saline (PBS) solution at pH 7.4. After a set incubation period, the weight of the hydrated gel was measured and compared against its initial weight. Excess water was blotted and removed before weighing. The swelling percentage was calculated using the following formula:

(1) $$\%Swelling=\frac{W_{s}-W_{d}}{W_{d}}\times 100$$

where *W_s_* is the weight of swollen gel and *W_d_* is the initial weight.

### 4.5. Colorimetric Response

To check the colorimetric response, a 10 × 10 × 2 mm^3^ piece of hydrogel was soaked in PBS with varying pH (4–8) at room temperature (25 °C). After 10 min, the color change was captured using a phone camera (iPhone 15, Apple Inc., Cupertino, CA, USA). RGB and grayscale values were measured using ImageJ software (Version 1.54s), and the corresponding values were plotted versus pH change to assess semi-quantitative color change correlation.

### 4.6. Drug Release Profile

To evaluate the in vitro release profile of LVX under conditions mimicking both healthy and infected wounds, hydrogel patch samples were placed into individual well plates. Each well was filled with 5.0 mL of PBS adjusted to a specific pH (5.0, 7.4, or 8.0). At predetermined time intervals, 1.0 mL aliquots were withdrawn for analysis and immediately replaced with an equal volume of fresh buffer to maintain sink conditions. The concentration of the released drug was then quantified using a Shimadzu UV mini-1240 UV–Visible Spectrophotometer. Initial drug release kinetics were determined by modeling the calculated % cumulative drug released (*Q*) using the following equations [77]:

Higuchi Model:

(2) $$Q=k_{H}t^{\frac{1}{2}}$$

Korsmeyer–Peppas:

(3) $$Q=k_{KP}t^{n}$$

where *n* is the release exponent.

Peppas–Sahlin:

(4) $$Q=k_{1}t^{m}+k_{2}t^{2m}$$

where *m* = 0.43 for a sphere, *m* = 0.45 for a cylinder, and *m* = 0.5 for a thin film.

## Figures and Tables

**Table 1:** Kinetic parameters for LVX release from 3D-printed hydrogels with varying GO content fitted to Higuchi, Korsmeyer–Peppas, and Peppas–Sahlin models.

Sample	pH	Higuchi		Korsmeyer–Peppas			Peppas–Sahlin		
		*k_H_*	R^2^	*n*	*k_KP_*	R^2^	*k*_1_ (Fickian Term)	*k*_2_ (Relaxation Term)	R^2^
GO-0	5	22.18	0.949	0.69	16.92	0.993	6.84	7.97	0.998
	7.4	24.36	0.947	0.69	18.52	0.994	7.15	8.94	0.999
	8	26.68	0.932	0.63	22.84	0.985	11.23	7.9	0.999
GO-1	5	24.77	0.947	0.69	18.86	0.994	7.37	9.04	0.999
	7.4	26.96	0.944	0.69	20.48	0.995	7.93	9.87	0.999
	8	33.26	0.978	0.81	24.89	0.983	5.75	19.34	0.997
GO-2	5	26.07	0.947	0.69	19.92	0.995	7.82	9.47	0.999
	7.4	28.24	0.947	0.69	21.57	0.995	8.49	10.25	0.999
	8	34.69	0.978	0.81	25.99	0.983	5.91	20.14	0.997
GO-3	5	23.51	0.948	0.69	17.91	0.994	6.94	8.6	0.999
	7.4	25.66	0.944	0.69	19.52	0.994	7.42	9.47	0.999
	8	32.7	0.985	0.86	23.23	0.993	11.02	21.28	0.999

**Table 2:** Kinetic parameters for LVX release from 3D-printed hydrogels with varying macropores fitted to Higuchi, Korsmeyer–Peppas, and Peppas–Sahlin models.

Sample	pH	Higuchi		Korsmeyer–Peppas			Peppas–Sahlin		
		*k_H_*	R^2^	*n*	*k_KP_*	R^2^	*k*_1_ (Fickian Term)	*k*_2_ (Relaxation Term)	*R* ^2^
0 Pores	5	19.64	0.887	0.58	15.32	0.981	3.12	12.87	0.985
	7.4	22.18	0.901	0.62	16.45	0.984	4.85	11.23	0.989
	8	24.52	0.923	0.68	18.21	0.991	7.45	10.95	0.996
4 Pores	5	23.41	0.912	0.64	18.1	0.988	5.23	12.65	0.992
	7.4	26.85	0.935	0.69	20.35	0.993	6.89	13.54	0.998
	8	30.12	0.956	0.74	22.45	0.995	5.12	17.89	0.999
9 Pores	5	27.56	0.928	0.67	21.05	0.991	7.15	14.23	0.995
	7.4	32.48	0.961	0.78	23.65	0.994	2.15	22.45	0.998
	8	38.65	0.984	0.88	26.12	0.992	8.54	33.12	0.996
16 Pores	5	29.85	0.935	0.68	23.15	0.992	8.45	15.1	0.997
	7.4	34.15	0.965	0.82	24.85	0.993	2.45	28.65	0.998
	8	41.25	0.991	1.05	24.1	0.996	18.25	42.65	0.999

**Table 3:** 3D-printed theranostic hydrogel formulation.

Entry	Water (% *w*/*w*)	AAm (% *w*/*w*)	HEA (% *w*/*w*)	CMC (% *w*/*v*)	GO (ppm)
A	40	30	30	0	0
B	40	30	30	0	1
C	40	30	30	0.01	1
D	40	30	30	0.02	1
E	40	30	30	0.01	1
F	40	30	30	0.01	2
G	40	30	30	0.01	3

All entries were formulated with 0.01% *w*/*w* PEGDA, 0.25% *w*/*w* LAP, 0.004% *w*/*w* BCP, and 1000 ppm LVX.

## Data Availability

The original contributions presented in this study are included in the article/supplementary material. Further inquiries can be directed to the corresponding author.

## Supplementary Materials

The following supporting information can be downloaded at https://www.mdpi.com/article/10.3390/gels12050393/s1: Figure S1. 3D-printed hydrogels without BCP (left), and with BCP (right); Figure S2. Hydrogel green color value vs. pH; Figure S3. Hydrogel grayscale value vs. pH; Figure S4. 3D printing accuracy as a function of measured dimensions vs. nominal dimensions.

## Abbreviations

The following abbreviations are used in this manuscript:

DLP	Digital light processing
AAm	Acrylamide
HEA	2-Hydroxyethyl acrylate
PEGDA	Polyethylene glycol diacrylate
LAP	Lithium phenyl-2,4,6-trimethylbenzoylphosphinate
BCP	Bromocresol purple
LVX	Levofloxacin
CMC	Carboxymethyl cellulose
GO	Graphene oxide
PBS	Phosphate-buffered saline
