# Supplementary material for: Vat Photopolymerization-Fabricated Theranostic Hydrogels for Smart Wound Management
Source: Gels. 2026 May 2;12(5):393. doi: 10.3390/gels12050393 (PMC13205843; doi:10.3390/gels12050393)
Supplement: Supplementary file 1 [file gels-12-00393-s001.zip › gels-4248657-supplementary.pdf]

Vat Photopolymerization–Fabricated Theranostic Hydrogels for Smart Wound Management

Karl Albright Tiston, Laureen Ida Ballesteros, Jo Marie Venus Agad, Patrick Meracandayo, Karlos Mayo Silva, Toni Beth Lopez, Nadnudda Rodthongkum, Voravee P. Hoven, and Rigoberto Advincula

Supporting Information

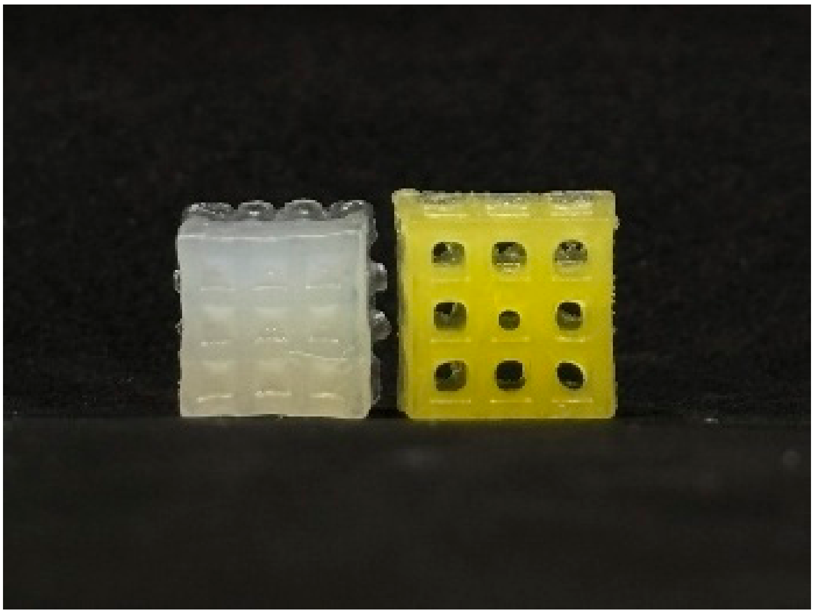

Figure S1. 3D Printed Hydrogels without BCP (left), and with BCP (right)

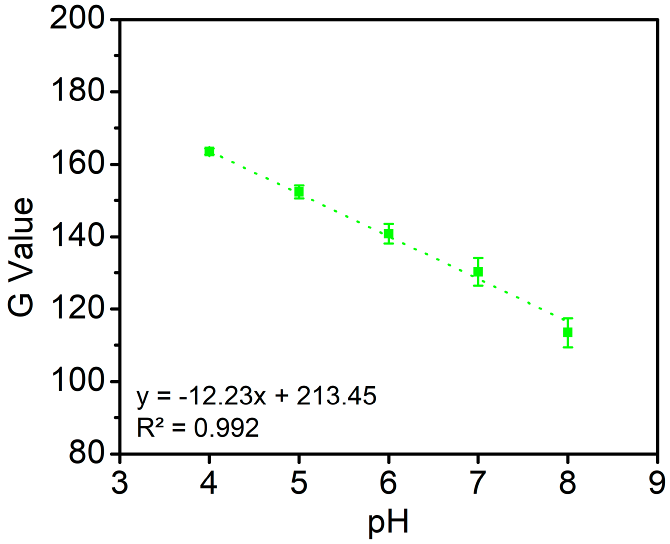

Figure S2. Hydrogel green color value vs. pH

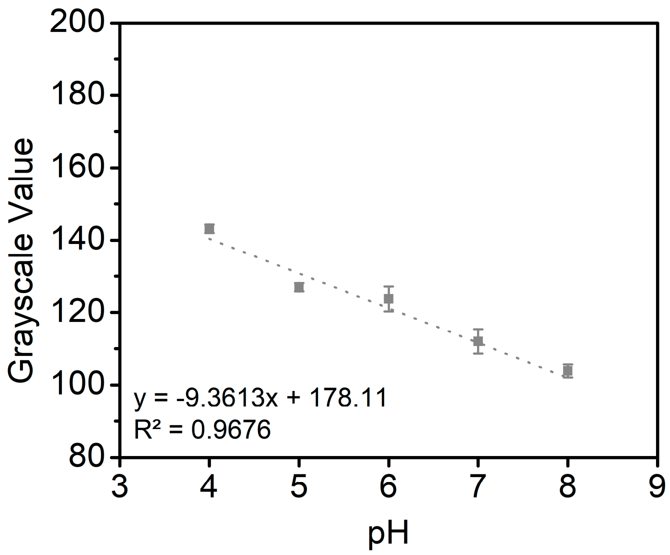

Figure S3. Hydrogel grayscale value vs. pH

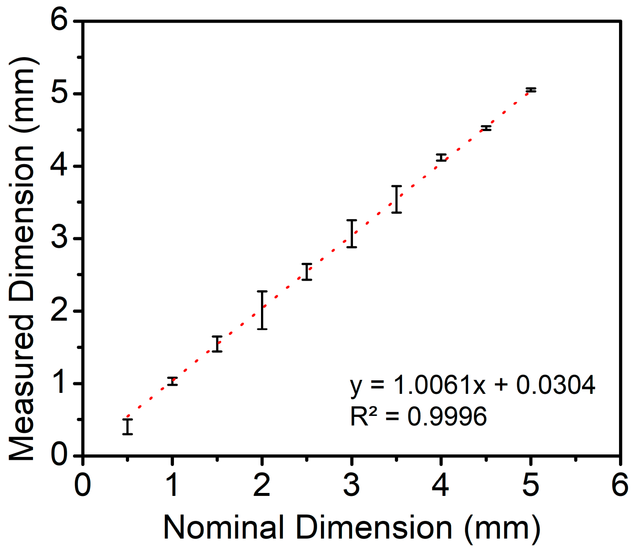

Figure S4. 3D Printing Accuracy as a function of measured dimensions vs. nominal dimensions
